# Bioconjugation of enzyme with silica microparticles: a promising platform for α-amylase partitioning†

**DOI:** 10.1039/c9ra02342a

**Published:** 2019-06-10

**Authors:** Maryam Karimi, Shiva Abdolrahimi, Gholamreza Pazuki

**Affiliations:** Chemical Engineering Department, Amirkabir University of Technology (Tehran Polytechnic) Tehran Iran ghpazuki@aut.ac.ir

## Abstract

Here, we report the implementation of α-amylase conjugated silica microparticles for improvement of α-amylase partitioning in a PEG–organic salt-based aqueous two phase system. A direct reduction method was employed for the synthesis of silica microparticles with simultaneous introduction of α-amylase. In this context, we synthesized three different silica α-amylase conjugated microparticles with variation of tetraethyl orthosilicate concentration, and thus the effect of final particle size and enzyme loading on partitioning was also studied. The partition coefficient ratio of α-amylase to Si:α-amylase of 2.186 : 21.701 validated an almost tenfold increase in separation. The microscopic structure of the system was thoroughly investigated in order to understand the extraction mechanism and any possible denaturation. Improved partition coefficients can be interpreted by the formation of α-amylase–silica–PEG carriers. Furthermore, circular dichroism (CD) spectra validated partial unfolding of the enzyme.

## Abstract 2

Here, we report the implementation of α-amylase conjugated silica microparticles for improvement of α-amylase partitioning in a PEG–organic salt-based aqueous two phase system.

## Graphical abstract

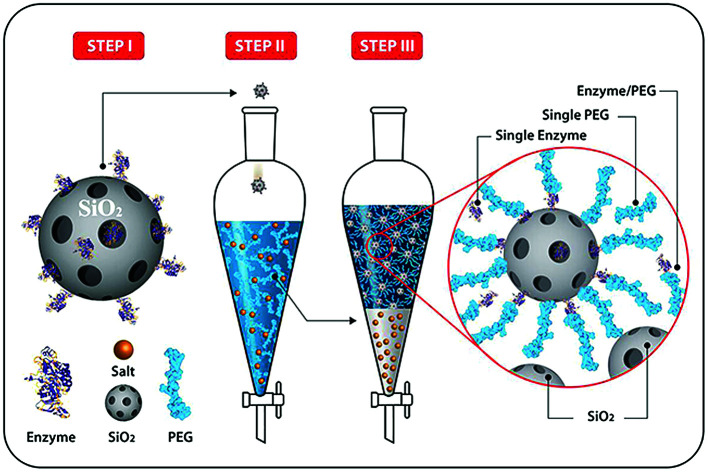

Enzymes as biomacromolecular proteins propose remarkable features for catalysis of metabolic pathways with high specificity and efficiency.^1,2^ New fields of application for enzymes are constantly growing. Among these, disease diagnostics, environmental monitoring, biomedical applications and immunosensing are emerging disciplines.^1,3^ Furthermore, recent evolution of modern biotechnology has induced rapid growth in the enzyme industry.^3^ On that account, high-yield isolation methods can play a crucial role in sustaining a low final cost of the product. Besides, the isolation method should not impose on the enzyme any conformational distortion and subsequent denaturation.^2^ In this regard, aqueous two phase systems (ATPS) propose gentle and biocompatible media for enzyme separation.^4–7^ The two immiscible phases are generally formed by the addition of structurally different polymers, a polymer and a salt, or two salts.^4,6,8^ Recently, many innovative research groups have focused on the partitioning of biomolecules in ATPS. Resultantly, polymer-based ATPS have been widely used for biomolecules’ separation and recovery.^9–14^

Hybridization of nanoparticles with biomolecules is an innovative research area which has pioneered various biological applications such as delivery, diagnosis, separation and imaging.^2,14–19^ Exceptional physiochemical properties of nanoparticles make them a suitable candidate for bioconjugation. Silica has been specially incorporated in this field due to its unique intrinsic features such as high surface area to volume ratio, adjustable surface chemistry and biocompatibility. Silica’s affinity toward various biomolecules can be modified by attachment of desired functional groups such as hydrophilic, hydrophobic, acidic or basic groups to the surface.^20–24^

A recent study reported the effect of bioconjugation of horseradish peroxidase to colloidal Au nanoparticles and subsequently the protein partitioning in ATPS was significantly improved.^25^ Furthermore, a recent pioneering research study described the effect of the introduction of silica nanoparticles in ATPS as an additive and significant enhancement of enzyme recovery was observed.^2^

In this communication, we report bioconjugation of α-amylase with silica in the process of synthesis of silica microparticles with a standard Stöber method.^26^ In this context, we studied the effect of variation of tetraethyl orthosilicate (TEOS) concentration on the synthesized particles. Moreover, we introduced the novel enzyme–microparticle carriers in conventional polyethylene glycol (PEG)–trisodium citrate (Na_3_ citrate) ATPS and to a notable degree partitioning was improved. For the synthesis of conjugated silica–α-amylase microparticles, a direct reduction method according to the Stöber method was employed. In this method, hydrolysis and condensation of TEOS in ethanol with the introduction of ammonium hydroxide was performed.^27^ α-Amylase along with TEOS was added to the starting solution comprising ethanol, water and ammonia. After sufficient stirring, the whitish precipitate was washed, centrifuged and dried. The produced particles are labelled Si:α-amylase for future reference (Fig. 1).

**Fig. 1:**
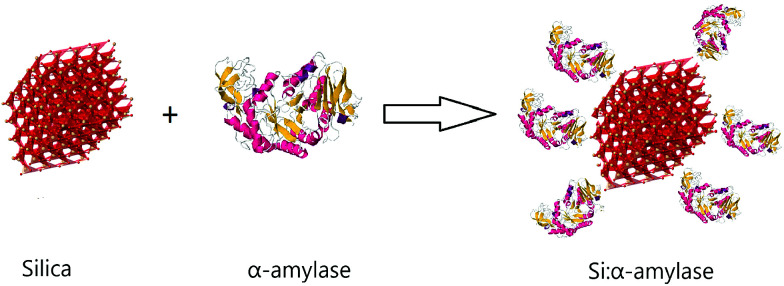
Schematic illustration of conjugated silica–α-amylase microparticles.

Using this scenario, we synthesized three different Si:α-amylase particles; however, characterization of these particles was required for thorough comprehension of the system. For this reason, FTIR spectroscopy was required to confirm conjugation in the synthesized Si:α-amylase particles. As can be inferred from Fig. 2b and c, the highest band at around 1100 cm^−1^ is due to Si–O–Si asymmetric stretching vibrations. The band at 470 cm^−1^ is associated with the Si–O–Si bending vibration. It can be interpreted, from the comparison of Fig. 2b and (c), that all the signature bands of silica are intact in the Si:α-amylase particles. Accordingly, no dramatic change in the silica structure is observed; however, the emergence of a new absorption band of the amide group at 1470 cm^−1^ (Fig. 2c) hints at the adsorption of α-amylase to the silica surface.

**Fig. 2:**
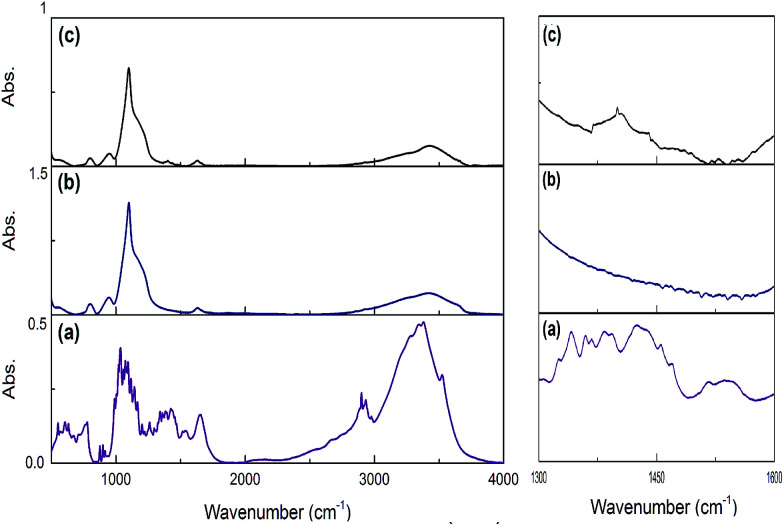
FTIR spectra of pure α-amylase, pure silica and α-amylase conjugated silica particles. (a) Pure α-amylase; (b) pure silica; (c) α-amylase conjugated silica particles.

Dynamic light scattering (DLS) was developed as an analytical tool for prediction of the hydrodynamic size of nanoparticles. In this study, the effect of TEOS concentration on the final particle size of Si:α-amylase was investigated. Additionally, the *Z*-average of the synthesized particles is tabulated in Table 1. Many research groups studied the effect of TEOS concentration on the final particle size of silica, and reported that the particle size increases with an increase in [TEOS] due to the increase in the concentration of primary particles in the induction period.^28^ Thus, it is speculated that increasing the amount of TEOS in the synthesis of Si:α-amylase particles leads to an increase in the final particle size. This is mainly due to the increase in the particle size of silica prior to conjugation with α-amylase.

**Table 1:** Size distribution of α-amylase conjugated silica particles with DLS

Particle	TEOS amount (ml)	*Z*-Average (nm)
Si:α-amylase1	1.55	557
Si:α-amylase2	9.97	1088
Si:α-amylase3	14.85	1374

Exact enzyme loading on the silica surface was estimated using TGA analysis (Fig. 3). Thus, the conjugated particles were placed in the chamber and they were exposed to temperature variation from 25 °C to 800 °C. A weight loss signature was observed in the range 60 °C to 170 °C which was assigned to the decomposition of α-amylase. Consequently, out of the total mass of the conjugated particles, 15.23, 13.9 and 13.78% was due to the α-amylase and the rest was the contribution of the silica particles. It can be inferred that the decrease in the final particle size is accompanied by the augmentation in the enzyme loading on the silica surface. This observation can be associated with the increase in the surface area, porosity and pore volume. In order to validate this assumption, Barrett–Joyner–Halenda (BJH) analysis was conducted. This method determines pore size distribution and specific pore volume.

**Fig. 3:**
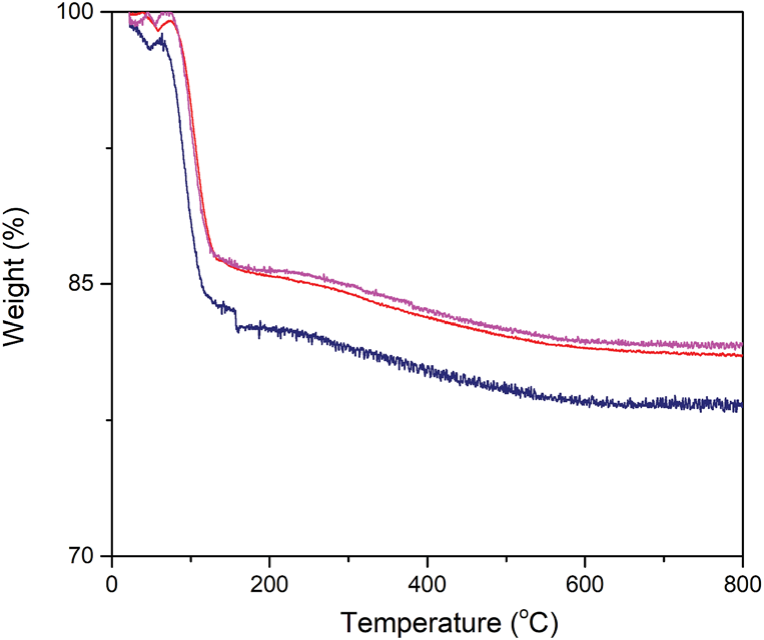
Thermogravimetric analysis of α-amylase conjugated silica particles: (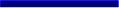) Si:α-amylase1; (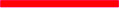) Si:α-amylase2; (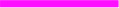) Si:α-amylase3.

As can be observed (Fig. 4), all of the synthesized particles have type II isotherms, which is characteristic of multilayer adsorption in non-porous solids. Accordingly, the pore size distribution of the synthesized particles was in the range of 1–155 nm. Moreover, Table 2 represents the subsequent specific surface area, pore size and pore volume. It is deduced that the smallest sized Si:α-amylase particle has the largest specific surface area and pore size. Consequently, reduction of Si:α-amylase particle size leads to the augmentation of pore volume, specific surface area and pore size. With an increase in these parameters, α-amylase has an amplified space to either position itself into the pores or the surface of the silica particles. Thus, the stated hypothesis was validated. As the enzyme molecules are non-spherical, some voids in the packing of the Si:α-amylase particles are exhibited; and resultantly a larger surface area and roughness is expected. The same observation was reported by Bhaskara Rao *et al.*^27^

**Fig. 4:**
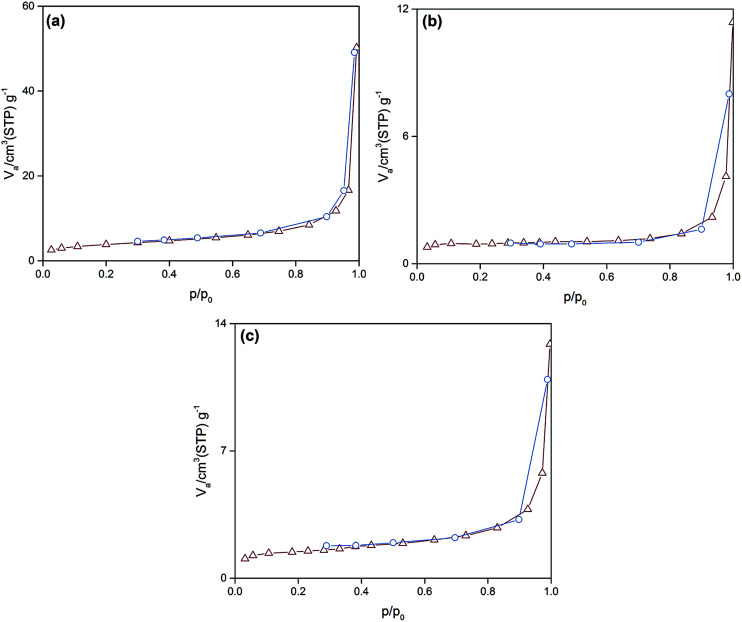
The N_2_ adsorption–desorption isotherm of (a) Si:α-amylase1; (b) Si:α-amylase2; (c) Si:α-amylase3 particles.

**Table 2:** Surface properties of synthesized silica–α-amylase particles

Particle	*V*_p_ (cm^3^ g^−1^)	*d*_p,peak_ (nm)	*a*_p_ (m^2^ g^−1^)
Si:α-amylase1	0.0702	68.93	9.5545
Si:α-amylase2	0.0157	21.53	2.3298
Si:α-amylase3	0.0127	24.79	1.1031

Industrial application of the enzyme requires thermal stability upon exposure to high operating temperatures. DSC analysis was performed for evaluation of the effect of temperature upon the activity and stability of α-amylase conjugated silica particles. Consequently, differences in the thermal behavior of α-amylase and Si:α-amylase particles are illustrated in Fig. 5. Comparatively, Si:α-amylase particles demonstrate less temperature variation with heat flow. This signifies that the presence of silica leads to an increase in heat capacity in Si:α-amylase. Resultantly, α-amylase manifests higher thermal stability upon conjugation.

**Fig. 5:**
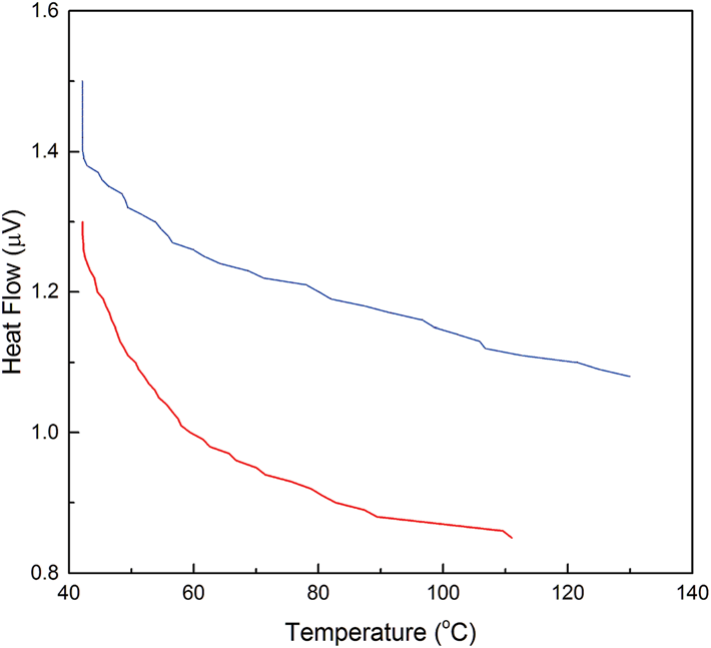
DSC analysis of α-amylase and α-amylase conjugated silica particles: (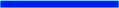) α-amylase; (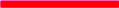) Si:α-amylase1.

Variation in TEOS concentration while synthesizing α-amylase conjugated silica particles gave rise to various particle sizes. Accordingly, its subsequent effect on α-amylase partitioning in PEG1000–Na_3_ citrate ATPS was evaluated. Besides, the feed’s initial concentration had a noticeable effect on the partitioning behavior of the biomolecule. For this reason, the effect of the initial concentration of phase forming components (PEG1000 and Na_3_ citrate) along with Si:α-amylase particles on partitioning of the enzyme was investigated. On that account, a Box–Behnken design was selected for the design of experiments and the respective partition coefficients (Table S3†) were evaluated. The partition coefficient of the biomolecule is defined as:1
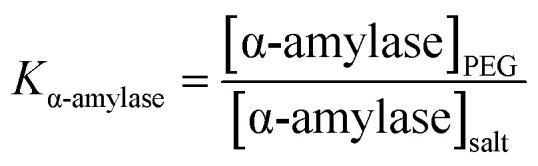
where [α-amylase]_PEG_ is the concentration of α-amylase in the PEG-rich phase and [α-amylase]_salt_ is its respective concentration in the salt-rich phase.

In this study, the smallest sized silica–α-amylase particle manifests the best recovery in terms of the partition coefficients. This could be interpreted from the TGA analysis which resulted in higher enzyme loading in Si:α-amylase1. For visual indication, a response contour plot for partitioning of Si-α-amylase1 is plotted in Fig. 6. The partition coefficients increase with the increase in the initial concentration of PEG1000. The increase in the initial concentration of PEG1000 makes the polymer-rich phase more hydrophobic. Consequently, it can be inferred that the extraction of the biomolecule is controlled by hydrophobic interactions. However, partition coefficients of the conjugated enzyme decrease with the increase in organic salt concentration. Increasing salt concentration causes water molecules to strongly bind to the salt. Consequently, a strong competition between the salt ions and enzyme molecules for water molecules forms, which results in a decrease in the enzyme solubility. Thus, the enzyme may segregate to the salt-rich phase.^29,30^ Besides, it is of great scientific importance to validate the improvement of the partition coefficients of α-amylase upon conjugation with silica microparticles. For this reason, the same experimental procedure was employed to estimate the partition coefficients of α-amylase in PEG-based ATPS (Table S2†). Consequently, the partition coefficient ratio of α-amylase to Si:α-amylase is 2.186 : 21.701 which represents a notable improvement in enzyme separation.

**Fig. 6:**
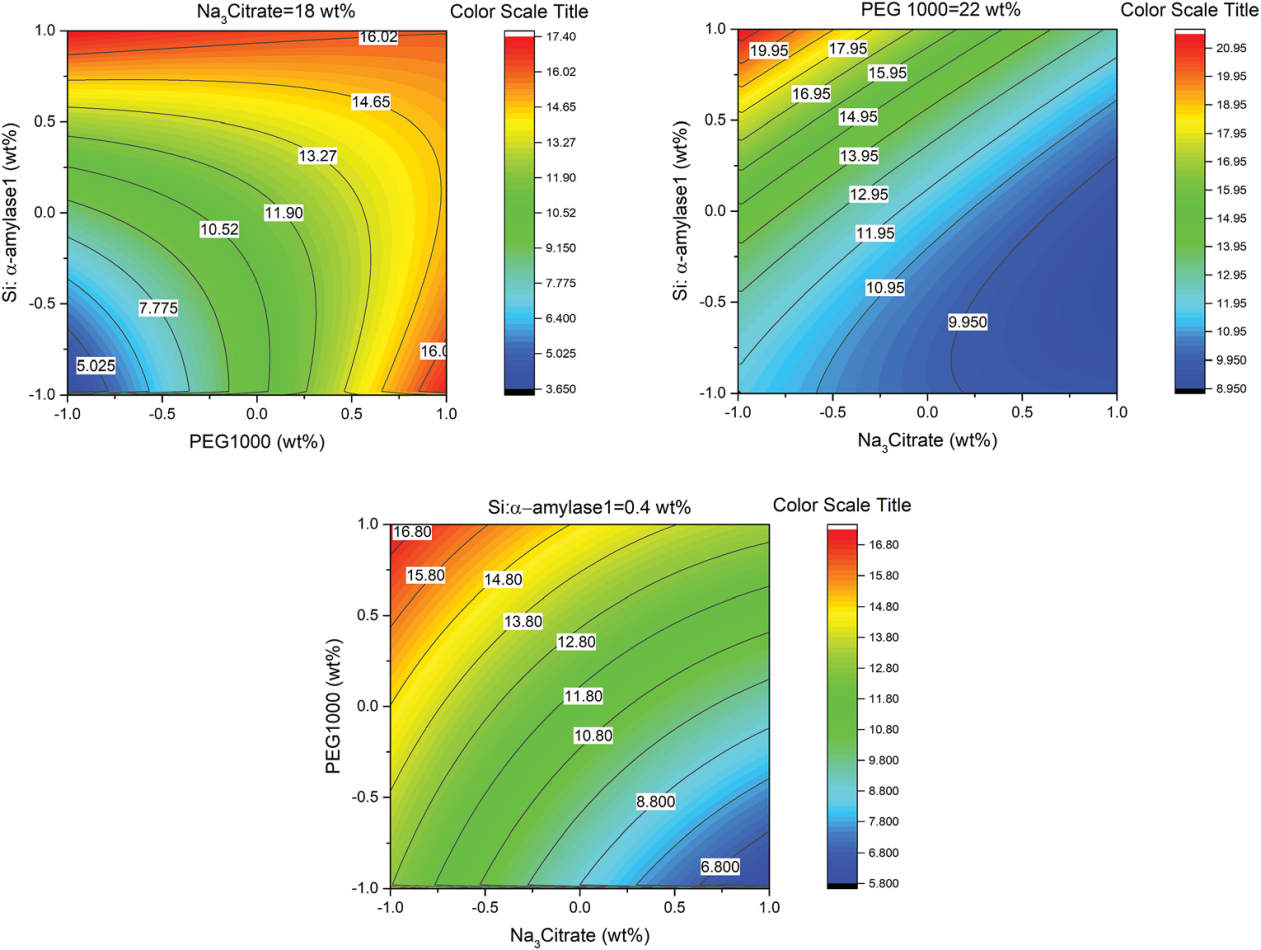
Response contour plots for partitioning of Si–α-amylase1 in PEG1000–Na_3_ citrate ATPS.

We applied FE-SEM for complete comprehension of the structure of the PEG-rich top phase. The aqueous two phase system was prepared and FESEM was performed after resting overnight. In the non-extractive (presence of no biomolecule) top phase (Fig. 7a), spherical particles of PEG with average size of 300 nm were spotted. However, the addition of the enzyme as the partitioning particle induced the detection of partially agglomerated α-amylase (Fig. 7b). It has been noted that a cysteine residue (C84) on the catalytic domain of α-amylase is mainly responsible for enzyme agglomeration.^31^ Besides, enzyme agglomeration can be obstructed by repulsive van der Waals and electrostatic energy barriers. Thus, ligation on the cysteine residue can be observed after proper sterical orientation of the enzyme with simultaneous conquest of the energy barriers. However, colloidal enzyme agglomerates are partially obstructed by the presence of PEG as the polymer chains circumvent α-amylase. Introduction of silica–α-amylase particles in ATPS leads to the presence of different particles. Partial removal of α-amylase from the conjugated particles led to the production of unconjugated enzyme and silica particles. Naturally, we would expect the appearance of enzyme–silica–polymer carriers (Fig. 7c). At our working pH, silica is hydrated and thus attachment of the positively charged PEG to the silanol groups is facilitated. On the other hand, α-amylase also adjoins the silica surface leading to the formation of α-amylase–silica–PEG carriers. Consequently, significant amelioration of the partition coefficient is validated.

**Fig. 7:**
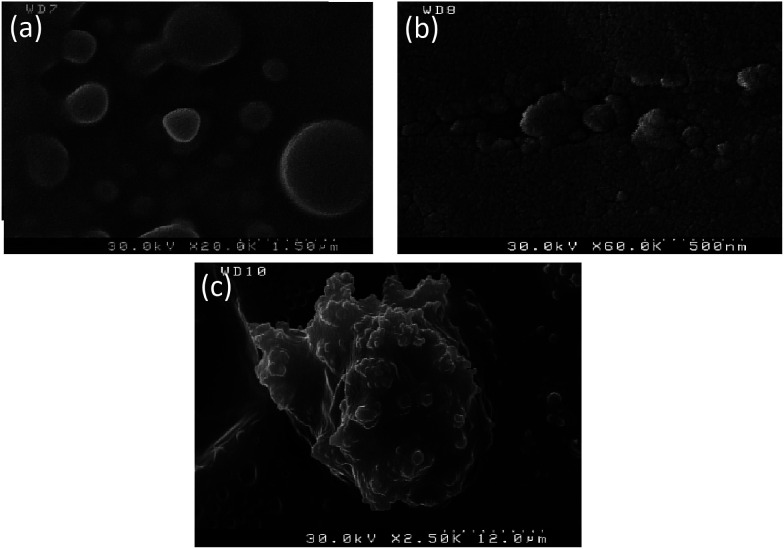
FE-SEM images: (a) PEG-rich upper phase without biomolecule, (b) α-amylase in the PEG-rich top phase, (c) silica–α-amylase particles in the top phase.

Recently, circular dichroism (CD) has been widely applied for characterization of the structure of enzymes. When enzymes are exposed to polarized light they show dichroism due to the amino acids and their ordered chiral structure. The secondary structure of the α-amylase before and after the conjugation can be speculated on due to the differential absorption of polarized light. Table 3 represents the CD spectra results. It is observed that α-amylase conjugation with silica led to partial unfolding of the enzyme.

**Table 3:** CD results of α-amylase and α-amylase conjugated silica

Secondary structure	α-Amylase (%)	Si:α-amylase (%)
α-Helix	1.90	0.3
β-Sheet	81.40	83.90
β-Turn	0	0
Random coil	16.70	15.80

In conclusion, we propose the synthesis of α-amylase conjugated silica microparticles for improvement of enzyme separation in PEG-based ATPS *via* the formation of α-amylase–silica–PEG carriers. Additionally, no denaturation was observed while partial unfolding of the enzyme was validated with CD. Furthermore, the graphical abstract demonstrates the main highlights of this research work.

## Conflicts of interest

There are no conflicts to declare.

## Supplementary Material

RA-009-C9RA02342A-s001
