# Supplementary material for: Bioconjugation of enzyme with silica microparticles: a promising platform for α-amylase partitioning
Source: RSC Adv. 2019 Jun 10;9(32):18217–21. doi: 10.1039/c9ra02342a (PMC9064637; doi:10.1039/c9ra02342a)
Supplement: RA-009-C9RA02342A-s001 [file RA-009-C9RA02342A-s001.pdf]

Electronic Supplementary Material (ESI) for RSC Advances.
This journal is © The Royal Society of Chemistry 2019

Supporting Information

Bioconjugation of Enzyme with Silica Microparticle: A Novel Platform for α-amylase Partitioning

M. Karimi, S.Abdolrahimi and G. R. Pazuki

Table S1 Experimental binodal curve mass fraction data for the system PEG 1000 (1) + Tri-sodium Citrate (2) + H_2O (3) at 298 K

w_1	w_2
0.4444	0.0613
0.4036	0.075
0.3756	0.0822
0.3445	0.0898
0.307	0.099
0.2807	0.1088
0.2502	0.1192
0.2216	0.1317
0.2062	0.1394
0.1777	0.1492
0.1582	0.1583
0.1424	0.1649
0.1275	0.1727
0.1068	0.1853
0.0937	0.1925
0.0856	0.1986
0.0737	0.2064
0.065	0.2196
0.055	0.2244
0.0544	0.2332
0.0475	0.2418
0.0461	0.2455

Table S2 Box-Behnken design matrix along with experimental response values for partitioning of α-amylase.

No.	α-amylase (gr)	PEG (wt%)	Salt (wt%)	Partition Coefficients
**1**	0.002	18	18	1.653
**2**	0.006	18	18	0.3136
**3**	0.006	22	22	0.243
**4**	0.004	26	22	0.803
**5**	0.004	22	18	1.669
**6**	0.006	22	14	2.1703
**7**	0.004	22	18	1.623
**8**	0.006	26	18	2.103
**9**	0.002	18	14	0.753
**10**	0.002	22	14	1.124
**11**	0.002	22	22	1.1443
**12**	0.004	18	22	0.646
**13**	0.004	26	18	1.325
**14**	0.004	22	18	1.865
**15**	0.004	26	22	0.808
**16**	0.004	26	14	2.186

Table S3 Box-Behnken design matrix along with experimental response values for partitioning of α-amylase conjugated silica particles.

No.	Si:α-amylase (gr)	PEG (wt%)	Salt (wt%)	Partition Coefficients		
				Si:α-amylase1	Si:α-amylase2	Si:α-amylase3
**1**	0.06	22	22	10.2351	7.0643	8.2436
**2**	0.04	22	18	11.0231	11.4821	7.9321
**3**	0.02	22	22	9.5209	6.0253	7.2351
**4**	0.02	26	18	15.2325	13.5565	11.7865
**5**	0.04	26	22	15.2365	10.9865	9.2365
**6**	0.06	22	14	21.7012	6.428	4.3253
**7**	0.04	18	14	10.8965	7.9254	8.9632
**8**	0.04	18	22	6.4610	5.4619	8.3401
**9**	0.02	22	14	14.2353	9.3656	7.8924
**10**	0.02	18	18	3.1365	2.5073	3.2321
**11**	0.06	26	18	15.9865	9.2564	7.2312
**12**	0.04	26	14	16.6898	12.3659	11.2352
**13**	0.06	18	18	19.4709	6.3423	7.2558
**14**	0.04	22	18	12.0521	10.2365	8.3401
**15**	0.04	22	18	11.5248	10.754	7.2345

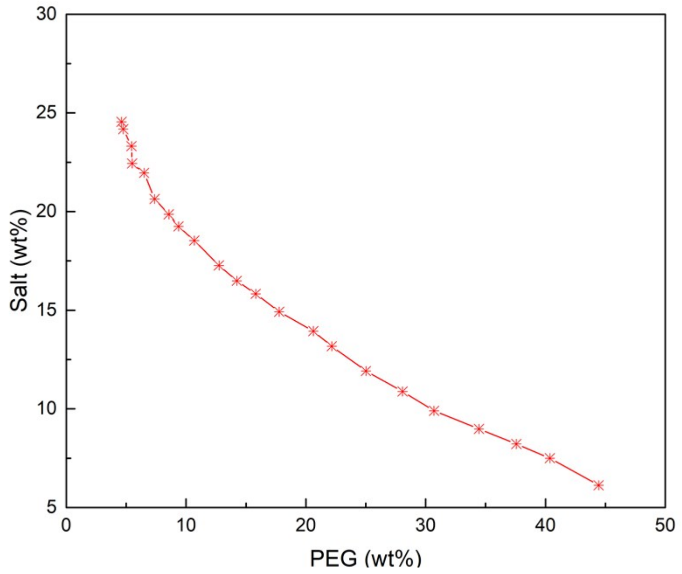

Figure S1 Ternary phase diagrams of PEG 1000 + Trisodium Citrate+ H_2O at 298 K and atmospheric pressure

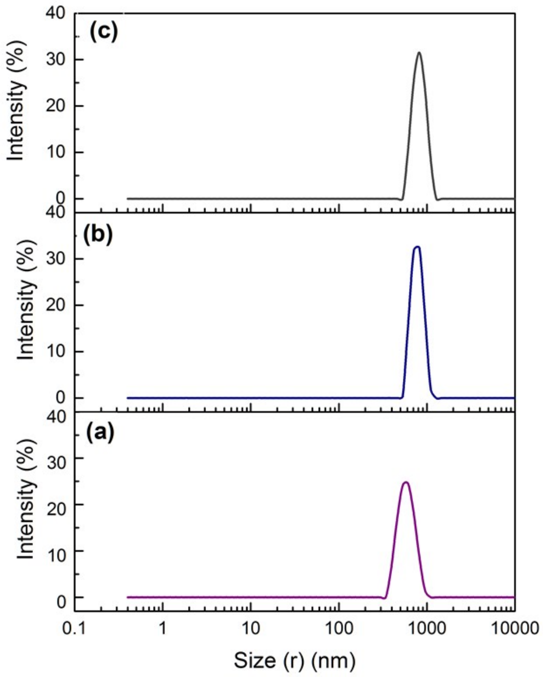

Figure S2 DLS graph (a) Si:α-amylase1, (b) Si:α-amylase2 and (c) Si:α-amylase3

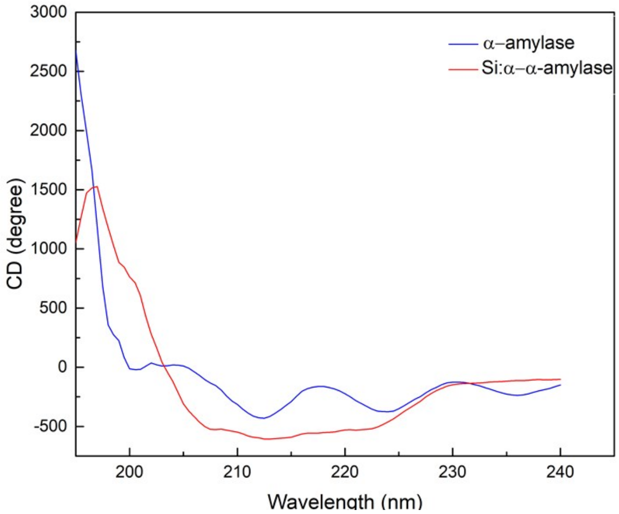

Figure S3 CD spectra of α-amylase and Si:α-amylase

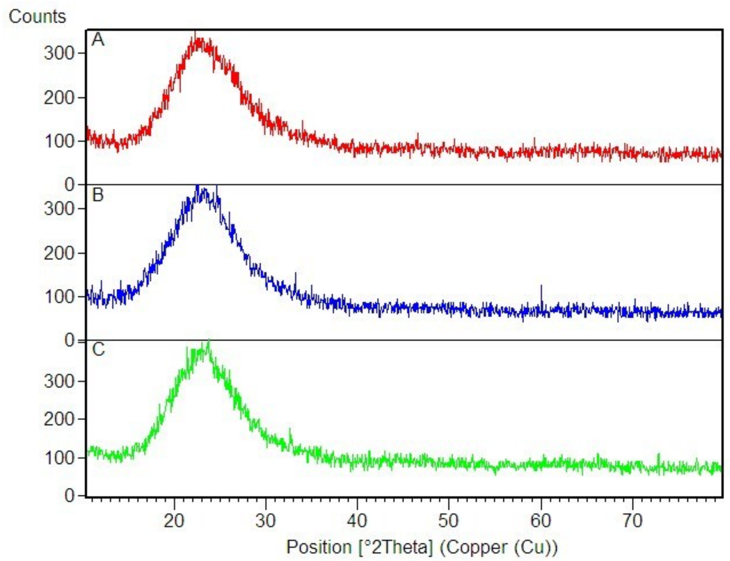

Figure S4 X-ray diffraction pattern of (A) Si:α-amylase1, (B) Si:α-amylase2 and (C) Si:α-amylase3
